# Effect of vitamin B12 replacement therapy in patients with premature ejaculation with B12 deficiency

**DOI:** 10.1186/s12894-026-02100-w

**Published:** 2026-02-27

**Authors:** Fatih Ustun, Emre Bulbul, Kadem Arslan, Nazlı Caner

**Affiliations:** 1 Department of Urology, Sultanbeyli State Hospital, Battalgazi, Pasaköy Ave No:60 Sultanbeyli, Istanbul, 34935 Türkiye; 2 Department of Urology, Vakfıkebir State Hospital, Trabzon, 61400 Türkiye; 3 Department of Internal Medicine, Sancaktepe Sehit Prof. Dr. Ilhan Varank Training and Research Hospital, University of Health Science, Istanbul, 34785 Türkiye; 4 Department of Clinical Biochemistry, Sultanbeyli State Hospital, Istanbul, 34935 Türkiye

**Keywords:** Intravaginal ejaculatory latency time, Premature ejaculation, Premature ejaculation diagnostic tool, Premature ejaculation profile, 5-hydroxytryptamine, Vitamin B12

## Abstract

**Background:**

Vitamin B12 plays a key role in the serotonergic pathway; we predicted that B12 replacement could be beneficial in the treatment of premature ejaculation (PE) in patients with B12 deficiency. We aimed to investigate the response to vitamin B12 replacement in PE patients with vitamin B12 deficiency.

**Methods:**

Between January 2025 and June 2025, a total of 152 individuals were included in this study, comprising 92 patients aged 18–45 who were diagnosed with PE and 60 healthy controls. The blood vitamin B12 levels of all participants were examined. Thirty-four patients with PE who presented with vitamin B12 deficiency were re-evaluated for ejaculation time after vitamin B12 replacement.

**Results:**

The mean blood vitamin B12 level was 237.8 ± 91.4 ng/L in the PE group and 297 ± 141.7 ng/L in the control group (*p* = 0.002). Following vitamin B12 replacement in the PE group with vitamin B12 deficiency, there was a statistically significant decrease in Premature Ejaculation Diagnostic Tool (PEDT) scores, a significant increase in Premature Ejaculation Profile (PEP) scores, and an increase in intravaginal latency time (*p* < 0.001). Of the patients diagnosed with PE who received B12 replacement therapy, 25 (73.5%) were satisfied with the treatment, while 9 (26.5%) were dissatisfied. Treatment satisfaction rates were 87.5% in those with lifelong PE and 61.1% in those with acquired PE (*p* = 0.082).

**Conclusion:**

In PE patients with B12 deficiency, normalizing blood B12 levels may be a viable treatment option. This is the first study to assess the effect of B12 replacement in PE patients with vitamin B12 deficiency.

**Supplementary Information:**

The online version contains supplementary material available at 10.1186/s12894-026-02100-w.

## Introduction

Premature ejaculation (PE) prevalence in society varies between 20% and 33%, depending on age, race, geography, and cultural values [1]. When the International Society for Sexual Medicine met for the second time in 2014 to define PE, the committee decided that lifelong PE was characterized by ejaculation that always or nearly always occurred before or within approximately 1 min of vaginal penetration, starting from the first sexual experience. The same committee defined acquired PE as a clinically significant and distressing reduction in intravaginal ejaculatory latency time (IELT), typically to approximately 3 min or less. In both cases, the inability to delay ejaculation in all or nearly all instances of vaginal penetration must be accompanied by distress, disappointment, and avoidance of sexual intercourse with the partner [2].

Ejaculation is defined as a complex physiological reflex involving emission and expulsion processes controlled by the neurological and hormonal systems. Any dysfunction in these pathways can lead to ejaculation disorders [3]. Although biological and psychological hypotheses have been suggested to explain the etiology and pathophysiology of PE, supporting data remain limited. Research has mainly focused on anxiety disorders, hypersensitivity of penile nerves, and 5-hydroxytryptamine (5-HT) receptor dysfunction [4–6]. There are hypotheses suggesting that neurotransmitters such as serotonin and dopamine play a key role in the pathophysiology of lifelong PE. In addition, studies have explored potential endocrinological and genetic factors [7]. In the etiology of acquired PE etiology, contributing factors may include sexual performance anxiety, relationship problems with the partner, prostatitis, erectile dysfunction, and thyroid hormone imbalances [8–10]. Among other risk factors are diabetes mellitus, insufficient physical activity, obesity, poor general health, stress and depressive mood, poor sleep quality, and vitamin D deficiency [11]. Vitamin B12 plays an important role in the metabolism of S-adenosylmethionine and tetrahydrobiopterin, which are intermediate products in the synthesis of 5-HT and nitric oxide. It acts as a methyl group donor in many methylation reactions. A deficiency in vitamin B12, which is involved in 5-HT metabolism, may disrupt neurotransmitter synthesis and indirectly affect ejaculation [12].

Serotonin, found in many regions of the brain, plays an important role at multiple levels of the nervous system. The active role of serotonin in ejaculation is considered to be mediated primarily by the 5-HT1A, 5-HT1B, and 5-HT2C receptor subtypes [13]. The European Association of Urology guidelines indicate that vitamin B12 deficiency is a risk factor for PE [11]. However, there is inadequate evidence in the literature regarding the effect of vitamin B12 replacement in these patients. Given the key role vitamin B12 plays in the serotonergic pathway, we hypothesized that vitamin B12 replacement could be beneficial in the treatment of patients with PE with vitamin B12 deficiency. The present study aimed to evaluate the outcomes of patients with PE with vitamin B12 deficiency before and after replacement therapy.

## Materials and methods

The local ethics committee approved this study (2024/381). All participants agreed to take part in this study. Informed consent was signed by all participants. The study included a total of 152 participants, comprising 92 patients aged 18–45 years diagnosed with PE and 60 healthy controls who visited the urology outpatient clinic between January 2025 and June 2025.

Both the PE group and the control group consisted of individuals aged 18–45 years who had been in a heterosexual relationship with a single partner for at least six months. The control group without PE included age-matched volunteers. The study did not include individuals with diabetes mellitus, renal failure, liver failure, chronic prostatitis, malignancy, thyroid disease, or pituitary gland disorders. Individuals with alcohol or substance dependence, any neurological disorder, or those receiving selective serotonin reuptake inhibitors, tricyclic antidepressants, or antipsychotics for a psychiatric disorder were also excluded.

Plasma vitamin B12 levels were tested in all participants. Venous blood samples were drawn between 8 and 10 a.m., after participants had fasted overnight for 10 to 12 h. Vitamin B12 levels were measured using the electrochemiluminescence immunoassay method on the Roche Cobas^®^ device in our laboratory. Vitamin B12 deficiency was defined as a level of < 197 ng/L, in accordance with the kit instructions. Patients were administered parenteral vitamin B12 treatment as indicated in the guidelines. 1000 mcg of vitamin B12 (cyanocobalamin) was administered intramuscularly to patients diagnosed with PE with VitB12 deficiency; every day for one week, then twice a week for two weeks, then once every two weeks [14, 15]. Participants diagnosed with PE who received vitamin B12 replacement therapy were given PEDT and PEP questionnaires, and their IELT results were recorded at the 3-month follow-up when their blood vitamin B12 levels reached the normal range. It takes time for vitamin B12 to be replenished at the tissue and cellular levels and for its functional effects to become apparent even with normal blood levels. Therefore, clinical follow-up is typically performed after 8–12 weeks [16].

Participants were evaluated using the Premature Ejaculation Diagnostic Tool (PEDT), Premature Ejaculation Profile (PEP), International Index of Erectile Function-Erectile Function (IIEF-EF), and Beck Depression Inventory (BDI) [17–20]. Patients with a PEDT score of ≥ 11 were diagnosed with PE [2]. Each participant completed the IIEF-EF questionnaire, and those with erectile dysfunction were excluded. Those with an IIEF-EF score ≥ 26 were included in the study [21]. Treatment satisfaction in patients with PE with vitamin B12 deficiency was evaluated using the Clinical Global Impression of Change (CGIC) questionnaire after B12 replacement. Patients with a CGIC score between 1 and 3 were defined as satisfied with treatment, while those with a score between 0 and − 3 were defined as dissatisfied [22, 23]. IELT was recorded by each patient using the stopwatch-measured technique [24].

### Statistical analysis

SPSS v. 25 was used for data analysis. The data distribution was tested by using the Kolmogorov-Smirnov test. Mean ± standard deviation was presented for normally distributed factors. The comparison of independent groups was tested by using the independent-samples t-test. The chi-squared test was used for categorical variables, and dependent groups were tested by using a paired-samples t-test. A *p*-value of < 0.05 was considered statistically significant. When the type 1 error is 0.05, and the test power is 0.8, the sample size should be at least 27 per group.

## Results

The mean age was 37.6 ± 8.3 years in the PE group and 36.1 ± 6.7 years in the control group (*p* = 0.106). There were no statistically significant differences between the two groups in terms of partner age (*p* = 0.219), duration of partnership (*p* = 0.178), frequency of sexual intercourse (*p* = 0.094), body mass index (*p* = 0.137), smoking (*p* = 0.864), IIEF-EF score (*p* = 0.068), or BDI score (*p* = 0.229). The mean PEDT score was 16.9 ± 3.1 in the PE group and 5.7 ± 2.1 in the control group (*p* < 0.001). The mean IELT was 64.8 ± 52.1 s in the PE group and 408.5 ± 219.5 s in the control group (*p* < 0.001). The mean blood vitamin B12 level was 237.8 ± 91.4 ng/L in the PE group and 297 ± 141.7 ng/L in the control group (*p* = 0.002). Table 1 presents the characteristics of the PE and control groups.

**Table 1:** Comparison of the characteristics of patients with PE and healthy controls

	PE group (*n* = 92)	Control group (*n* = 60)	*p*-value
Age (years), mean ± SD	37.6 ± 8.3	36.1 ± 6.7	0.106^a^
Partner age (years), mean ± SD	34.6 ± 6.4	32.9 ± 5.9	0.219^a^
Duration of partnership (years), mean ± SD	12.4 ± 7.8	10.4 ± 7.7	0.178^a^
Frequency of intercourse (per month), mean ± SD	7.9 ± 3.3	8.7 ± 2.6	0.094^a^
Smoker, n (%)	51 (55.4%)	35 (58.3%)	0.864^b^
BMI (kg/m^2^), mean ± SD	26.7 ± 3.8	27.2 ± 4.6	0.137^a^
IELT (seconds), mean ± SD	64.8 ± 52.1	408.5 ± 219.5	< 0.001^a^
PEDT score, mean ± SD	16.9 ± 3.1	5.7 ± 2.1	< 0.001^a^
PEP score, mean ± SD	4.7 ± 2.6	13.7 ± 1.7	< 0.001^a^
IIEF-EF score, mean ± SD	26.4 ± 3.1	27.2 ± 1.9	0.068^a^
BDI, mean ± SD	7.5 ± 3.6	6.8 ± 3.3	0.229^a^
Vitamin B12 (ng/L), mean ± SD	237.8 ± 91.4	297.2 ± 141.7	0.002

^a^Independent-samples t-test; ^b^Chi-squared test

*PE* premature ejaculation (PEDT score ≥ 11), *SD* standard deviation, *BMI* body mass index, *IELT* intravaginal ejaculatory latency time, *PEDT* Premature Ejaculation Diagnostic Tool, *PEP* Premature Ejaculation Profile, *IIEF-EF* International Index of Erectile Function-Erectile Function, *BDI* Beck Depression Inventory

Among patients with PE, 48 (52.2%) had lifelong PE, while 44 (47.8%) had acquired PE. The mean IELT was 47.2 ± 32.6 s in the lifelong PE group and 83.9 ± 62.1 s in the acquired PE group (*p* = 0.004), and the mean PEDT score was 17.8 ± 2.3 and 15.9 ± 3.4, respectively (*p* = 0.012). There were no statistically significant differences between the lifelong and acquired PE groups in terms of age (*p* = 0.234), body mass index (*p* = 0.561), or vitamin B12 levels (*p* = 0.159) (Table 2).

**Table 2:** Association between PE type and patients’ characteristics

	Life-long PE (*n* = 48)	Acquired PE (*n* = 44)	*p*-value
Age (years), mean ± SD	36.7 ± 7.3	39.6 ± 9.1	0.234^a^
Partner age (years), mean ± SD	33.8 ± 6.9	35.6 ± 5.7	0.241^a^
Duration of partnership (years), mean ± SD	11.3 ± 8.2	13.8 ± 7.1	0.151^b^
Frequency of intercourse (month), mean ± SD	7.9 ± 3.2	7.8 ± 3.4	0.931^a^
BMI (kg/m^2^), mean ± SD	26.4 ± 4.1	26.9 ± 3.4	0.561^a^
Smoker, n (%)	28 (58.3%)	23 (52.3%)	0.930^b^
IIEF-EF score, mean ± SD	26.4 ± 3.5	26.3 ± 2.6	0.523^a^
IELT (seconds), mean ± SD	47.2 ± 32.6	83.9 ± 62.1	0.004^a^
PEDT score, mean ± SD	17.8 ± 2.3	15.9 ± 3.4	0.012^a^
PEP score, mean ± SD	4.3 ± 2.4	5.3 ± 2.8	0.072^a^
BDI, mean ± SD	8.2 ± 3.6	6.9 ± 3.6	0.250^a^
Vitamin B12 (ng/L), mean ± SD	247.8 ± 87.4	226.8 ± 95.3	0.159^a^

^a^Independent-samples t-test; ^b^Chi-squared test

*PE* premature ejaculation (PEDT score ≥ 11), *SD* standard deviation, *BMI* Body mass index, *IELT* intravaginal ejaculatory latency time, *PEDT* Premature Ejaculation Diagnostic Tool, *PEP* Premature Ejaculation Profile, *IIEF-EF* International Index of Erectile Function-Erectile Function, *BDI* Beck Depression Inventory

Vitamin B12 deficiency was detected in 49 (53.2%) of patients with PE. Follow-up data could not be obtained for 15 patients during this process, resulting in their exclusion from follow-up. Pre- and post-vitamin B12 replacement therapy evaluations were therefore conducted in 34 patients with PE with vitamin B12 deficiency. There is no statistically significant difference between the patients with follow-up and those with loss to follow-up (Table 3).

**Table 3:** Characteristics of patients being followed and those with loss to follow-up

	Follow-up group	Loss of follow-up group	*p*-value
PEDT score	16.7 ± 3.1	17.1 ± 3.4	0.732^a^
PEP score	4.7 ± 2.8	4.3 ± 2.9	0.691^a^
Vitamin B12 (ng/L)	179.3 ± 18.1	171.2 ± 26.3	0.348^a^
IELT (seconds)	74.1 ± 60.1	91.4 ± 60.9	0.208^a^

^a^Independent-samples t-test

*PEDT* Premature Ejaculation Diagnostic Tool, *PEP* Premature Ejaculation Profile, *IELT* intravaginal ejaculatory latency time

In the PE group with vitamin B12 deficiency, a statistically significant reduction in PEDT scores and an increase in PEP scores and IELT were observed after vitamin B12 replacement therapy (*p* < 0.001) (Table 4). Twenty-five (73.5%) patients were satisfied with replacement therapy, while 9 (26.5%) were dissatisfied. Among patients with vitamin B12 deficiency, 87.5% (*n* = 14/16) with lifelong PE and 61.1% (*n* = 11/18) with acquired PE reported satisfaction with treatment after vitamin B12 replacement therapy (*p* = 0.082) (Table 5).

**Table 4:** Effect of vitamin B12 replacement therapy in patients with PE with B12 deficiency

	Before B12 replacement	After B12 replacement	*p*-value
PEDT score	16.7 **±** 3.1	10.9 **±** 4.3	< 0.001^a^
PEP score	4.7 **±** 2.8	9.1 **±** 3.8	< 0.001^a^
Vitamin B12 (ng/L)	179.3 **±** 18.1	524.1 **±** 179.6	< 0.001^a^
IELT (seconds)	74.1 **±** 60.1	135.3 **±** 110.8	< 0.001^a^

^a^Paired-samples t-test. Data are presented as mean ± standard deviation

*PE* Premature ejaculation (PEDT score ≥ 11), *PEDT* Premature Ejaculation Diagnostic Tool, *PEP* Premature Ejaculation Profile, *IELT* intravaginal ejaculatory latency time

**Table 5:** Treatment satisfaction after vitamin B12 replacement according to PE type

	Life-long PE (*n* = 16)	Acquired PE (*n* = 18)	*p*-value
Satisfied, n (%)	14 (87.5%)	11 (61.1%)	0.082^a^
CGIC score: +1, n (%)	9 (56%)	3 (16.6%)	
CGIC score: +2, n (%)	4 (25%)	3 (16.6%)	
CGIC score: +3, n (%)	1 (6.2%)	5 (27.7%)	
Dissatisfied, n (%)	2 (12.5%)	7 (38.9%)	

^a^Chi-squared test

*PE* premature ejaculation (Premature Ejaculation Diagnostic Tool score ≥ 11), *CGIC* Clinical Global Impression of Change

## Discussion

Vitamin B12 deficiency affects serotonin metabolism and may disrupt the physiology of ejaculation [12]. In the present study, the response to vitamin B12 replacement therapy in patients with PE with vitamin B12 deficiency was examined in detail. After vitamin B12 replacement therapy, a decrease in PEDT scores was observed in the PE group with vitamin B12 deficiency. Moreover, there was a statistically significant increase in PEP scores and intravaginal latency time. This suggests that normalizing blood B12 levels may be a suitable option for PE treatment among patients with B12 deficiency.

The literature indicates that vitamin B12 levels in patients with PE are lower than in healthy controls without PE [25]. In this study, blood vitamin B12 levels were also lower in the PE group compared to the healthy control group. Sonbahar and Culha reported that the efficacy of dapoxetine treatment in 62 patients with PE decreased in parallel with a reduction in vitamin B12 levels [23].

Vitamin B12 is a significant cofactor in the metabolism of S-adenosylmethionine, an intermediate in the synthesis of serotonin. This methyl donor role is essential for many methylation reactions in the brain and for serotonin metabolism. Serotonin, found in the hypothalamus, brainstem, and spinal cord, plays a key role in regulating ejaculation. Studies examining the relationship between 5-HT and ejaculation by investigating brain function have been conducted and have yielded similar results. Following microinjection of 5-HT into the serotonergic projection area within the medial preoptic area and the forebrain, an increase in the ejaculation latency period was observed in rats. Similarly, after administering 5-HT to the raphe nuclei containing the serotonergic cell bodies of male rats, a facilitation of ejaculation behaviour was observed [26]. Therefore, although vitamin B12 does not directly affect the ejaculation process, its role as a cofactor in serotonin metabolism is crucial [12]. It is stated that one of the causes of PE may be low serum vitamin B12 levels. Gokcen and Gokcen reported that patients with chronic gastritis and vitamin B12 deficiency had significantly shorter IELT and significantly higher PEDT scores compared to the healthy individuals [27].

Ejaculation is a two-phase complex reflex involving the central and peripheral nervous systems and various neurotransmitters [3]. Stimulation of postsynaptic 5-HT1B or 5-HT2C receptors delays ejaculation, whereas activation of presynaptic 5-HT1A receptors inhibits serotonin release and shortens ejaculation [13]. Vitamin B12 prevents Wallerian degeneration, which causes peripheral neuropathy. It is one of the factors that prevent the onset of peripheral neuropathy by contributing to nerve cell regeneration and remyelination. Vitamin B12, which plays a role in nerve regeneration, also supports the protection of myelin sheaths. As a result of all this, improvement in penile nerve function and nerve conduction velocity is achieved [28].

In this study, among patients with PE who received replacement therapy for vitamin B12 deficiency, treatment satisfaction was higher in those with lifelong PE compared to those with acquired PE (87.5% vs. 61.1%), although this difference was not statistically significant. In the etiology of lifelong PE, serotonergic mechanisms involving vitamin B12 as a cofactor may play a crucial role. In light of the findings outlined above, there is a potential clinical benefit to test blood vitamin B12 levels in patients presenting with PE, and additionally, prioritizing replacement therapy for B12-deficient patients, especially those with lifelong PE.

There are several limitations, such as a small sample size and a short follow-up. Additionally, lack of a placebo control group, insufficient mechanistic assessment (no direct detection of 5-HT-related indicators), and single-center design were other limitations. The results demonstrate an association rather than definitive therapeutic efficacy. However, considering the limited data published on this topic, we believe that our findings, particularly indicating the benefit of vitamin B12 replacement for patients with PE with vitamin B12 deficiency, will contribute to the existing literature. Further multi-center, large-sample randomized controlled trials with placebo controls, long-term follow-up, and molecular mechanism verification are certainly needed to clarify the effectiveness of this treatment.

## Conclusions

In patients with PE presenting with vitamin B12 deficiency, there was a statistically significant decrease in PEDT scores and an increase in PEP scores, and IELT were observed after B12 replacement. Normalizing blood B12 levels in this patient population may be a viable option for PE treatment. This is the first study to investigate the effect of B12 replacement in PE patients with vitamin B12 deficiency.

## Supplementary Information

Supplementary Material 1.

Supplementary Material 2.

## Data Availability

The datasets analysed during the current study are not publicly available but are accessible from the corresponding author Fatih Ustun on reasonable request.

## Abbreviations

PE: Premature ejaculation
PEDT: Premature Ejaculation Diagnostic Tool
PEP: Premature Ejaculation Profile
IELT: Intravaginal Ejaculatory Latency Time
5-HT: 5-hydroxytryptamine
IIEF-EF: International Index of Erectile Function-Erectile Function
BDI: Beck Depression Inventory
CGIC: Clinical Global Impression of Change

## References

[1] Jannini EA, Lenzi A. Ejaculatory disorders: epidemiology and current approaches to definition, classification and subtyping. World J Urol. 2005;23:68–75. 10.1007/s00345-004-0486-9. 15902473 10.1007/s00345-004-0486-9

[2] Serefoglu EC, McMahon CG, Waldinger MD, Althof SE, Shindel A, Adaikan G, et al. An evidence-based unified definition of lifelong and acquired premature ejaculation: report of the second international society for sexual medicine ad hoc committee for the definition of premature ejaculation. Sex Med. 2014;2:41–59. 10.1002/sm2.27. 25356301 10.1002/sm2.27 PMC4184676

[3] Clement P, Guiliano F. Physiology and Pharmacology of Ejaculation. Basic Clin Pharmacol Toxicol. 2016;119(3):18–25. 10.1111/bcpt.12546. 26709195 10.1111/bcpt.12546

[4] Ventus D, Gunst A, Karna A, Jern P. No Evidence for Long-Term Causal Associations Between Symptoms of Premature Ejaculation and Symptoms of Anxiety, Depression, and Sexual Distress in a Large, Population-Based Longitudinal Sample. J Sex Res. 2017;54(2):264–72. 10.1080/00224499.2016.1255301. 27982691 10.1080/00224499.2016.1255301

[5] Sun Z, Liao Z, Zheng Q, Chen J, Lv B, Bao C, et al. A Study of Differences in Penile Dorsal Nerve Somatosensory Evoked Potential Testing Among Healthy Controls and Patients With Primary and Secondary Premature Ejaculation. J Sex Med. 2021;18(4):732–6. 10.1016/j.jsxm.2021.01.186. 33744179 10.1016/j.jsxm.2021.01.186

[6] Khan HL, Bhatti S, Abbas S, Khan YL, Gonzalez RMM, Aslamkhan M et al. Serotonin transporter (5-HTTLPR) genotypes and trinucleotide repeats of androgen receptor exert a combinatorial effect on hormonal milieu in patients with lifelong premature ejaculation. Andrology.2018;6(6):916–926. 10.1111/andr.12518 10.1111/andr.12518 30019487

[7] Waldinger MD. The pathophysiology of lifelong premature ejaculation. Transl Androl Urol. 2016;5(4):424–33. 10.21037/tau.2016.06.04. 27652215 10.21037/tau.2016.06.04 PMC5001987

[8] Carani C, Isidori AM, Granata A, Carosa E, Maggi M, Lenzi A, et al. Multicenter study on the prevalence of sexual symptoms in male hypo- and hyperthyroid patients. J Clin Endocrinol Metab. 2005;90(12):6472–9. 10.1210/jc.2005-1135. 16204360 10.1210/jc.2005-1135

[9] Corona G, Petrona L, Mannucci E, Jannini EA, Mansani R, Magini A, et al. Psycho-biological correlates of rapid ejaculation in patients attending an andrologic unit for sexual dysfunctions. Eur Urol. 2004;46(5):615–22. 10.1016/j.eururo.2004.07.001. 15474272 10.1016/j.eururo.2004.07.001

[10] McMahon CG, Jannini EA, Serefoglu EC, Hellstrom WJG. The pathophysiology of acquired premature ejaculation. Transl Androl Urol. 2016;5(4):434–49. 10.21037/tau.2016.07.06. 27652216 10.21037/tau.2016.07.06 PMC5001985

[11] Salonia A, Boeri L, Capogrosso P, Corona G, Dinkelman-Smith M, Falcone M et al. EAU Guidelines on Sexual and reproductive health 2025 Update. Page: 58. https://uroweb.org/guidelines/sexual-and-reproductive-health/chapter/disorders-of-ejaculation

[12] Bottiglieri T. Folate, vitamin B12, and S-adenosylmethionine. Psychiatr Clin North Am. 2023;26(1):1–13. 10.1016/j.psc.2012.12.001. 10.1016/j.psc.2012.12.001 23538072

[13] Giuliano F. 5-Hydroxytryptamine in premature ejaculation: opportunities for therapeutic intervention. Trends Neurosci. 2007;30(2):74–84. 10.1016/j.tins.2006.12.002. 10.1016/j.tins.2006.12.002 17169440

[14] Shipton MJ, Thachil J. Vit B12 deficiency - A 21st century perpective. Clin Med (Lond). 2015;15(2):145–50. 10.7861/clinmedicine.15-2-145. 25824066 10.7861/clinmedicine.15-2-145 PMC4953733

[15] Turkish Haematology Association B12 Vitamin Deficiency Diagnosis and Treatment Guidelines Edition. 2011, P:6 https://www.thd.org.tr/thddata/books/94/bolum-i-b12-vitaminieksikligi-tani-ve-tedavi-kilavuzu

[16] Vitamin B. deficiency in over 16s: diagnosis and management. London: National Institute for Health and Care Excellence (NICE); 06 March 2024. https://www.nice.org.uk/guidance/ng239 38713783

[17] Symonds T, Perelman MA, Althof S, Giuliano F, Martin M, May K, et al. Development and validation of a premature ejaculation diagnostic tool. Eur Urol. 2007;52:565–73. 10.1016/j.eururo.2007.01.028. 17275165 10.1016/j.eururo.2007.01.028

[18] Patrick DL, Giuliano F, Ho KF, Gagnon DD, McNulty P, Rothman M. The Premature Ejaculation Profile: Validation of self-reported outcome measures for research and practice. BJU Int. 2009;103:358–64. 10.1111/j.1464-410X.2008.08041.x. 18793300 10.1111/j.1464-410X.2008.08041.x

[19] Waldinger MD, Quinn P, Dilleen M, Mundayat R, Schweitzer DH, Boolell M. A multinational population survey of intravaginal ejaculation latency time. J Sex Med. 2005;2(4):492–7. 10.1111/j.1743-6109.2005.00070.x. 16422843 10.1111/j.1743-6109.2005.00070.x

[20] Beck AT, Steer RA, Garbin MG. Psychometric properties of the Beck Depression Inventory: Twenty-five years of evaluation. Clin Psychol Rev. 1988;8:77–100.

[21] Cappelleri JC, Rosen RC, Smith MD, Mishra A, Osterloh IH. Diagnostic evaluation of the erectile function domain of the International Index of Erectile Function. Urology. 1999;54(2):346–51. 10.1016/s0090-4295(99)00099-0. 10443736 10.1016/s0090-4295(99)00099-0

[22] Althof SE, Brock GB, Rosen RC, Rowland DL, Aquilina JW, Rothman M, et al. Validity of the patient-reported clinical global impression of change as a measure of treatment reponse in men with premature ejaculation. J Sex Med. 2010;7:2243–52. 10.1111/j.1743-6109.2010.01793.x. 20367770 10.1111/j.1743-6109.2010.01793.x

[23] Sonbahar AE, Culha MG. Effect of serum Vit B12 levels on premature ejaculation. J Coll Physicians Surg Pak. 2024;34(3):351–4. 10.29271/jcpsp.2024.03.351. 38462874 10.29271/jcpsp.2024.03.351

[24] Salonia A, Bettocchi C, Boeri L, Capogrosso P, Carvalho J, Cilesiz NC, et al. EAU Working Group on Male Sexual and Reproductive Health. European Association of Urology Guidelines on Sexual and Reproductive Health-2021 Update: Male Sexual Dysfunction. Eur Urol. 2021;80:333–57. 10.1016/j.eururo.2021.06.007. 34183196 10.1016/j.eururo.2021.06.007

[25] Pazır Y, Guler H, Bulut TB, Ari E, Aktas S, Kadıhasanoglu M. The association of reproductive hormones, thyroid function, and vitamin levels with premature ejaculation: A prospective case-control study. Investig Clin Urol. 2024;65(2):173–9. 10.4111/icu.20230213. 38454827 10.4111/icu.20230213 PMC10925740

[26] Giuliano F, Clément P. Serotonin and premature ejaculation: from physiology to patient management. Eur Urol. 2006;50:454–66. 10.1016/j.eururo.2006.05.055. 16844284 10.1016/j.eururo.2006.05.055

[27] Gökçen K, Gökçen P. Effects of Vitamin B12 Deficiency on Ejaculation Time in Patients with Chronic Gastritis. J Urol Surg. 2019;6(3):244–51. 10.4274/jus.galenos.2019.2591.

[28] Baltrusch S. The role of Neurotropic B Vitamins in nerve regeneration. Biomed Res Int. 2021;202113:9968228. 10.1155/2021/9968228. 10.1155/2021/9968228 PMC8294980 34337067

