# Supplementary material for: Effect of vitamin B12 replacement therapy in patients with premature ejaculation with B12 deficiency
Source: BMC Urol. 2026 Feb 27;26:90. doi: 10.1186/s12894-026-02100-w (PMC13049710; doi:10.1186/s12894-026-02100-w)
Supplement: Supplementary file 1 — Supplementary Material 1. [file 12894_2026_2100_MOESM1_ESM.pdf]

EK-2

S.B.Ü. SANCAKTEPE ŞEHİT PROF. DR. İLHAN VARANK E.A.H.
BİLİMSEL ARAŞTIRMALAR ETİK KURULU KARAR FORMU

ARAŞTIRMANIN AÇIK ADI		Vitamin B12 eksikliği olan prematür ejakülasyon hastalarında vitamin B12 replasmanı öncesi ve sonrası prematür ejakülasyonun değerlendirilmesi
ETİK KURUL BAŞVURU DOSYA NO-TARİH	Dosya No: 381	Tarih: 25.12.2024
**ETİK KURUL BİLGİLERİ**	ETİK KURUL ADI	Sancaktepe Şehit Profesör İlhan Varank Eğitim ve Araştırma Hastanesi Bilimsel Araştırmalar Etik Kurulu
	AÇIK ADRESİ	Emek, Sarıgazi Emek, Namık Kemal Cd. No:7 Sancaktepe/İstanbul
	TELEFON	(0216) 606 33 00
	E-POSTA	sancaktepeetikkurul@gmail.com
**BAŞVURU BİLGİLERİ**	ARAŞTIRMACI UNVANI ADI SOYADI	Uzm. Dr. Fatih ÜSTÜN
	UZMANLIK ALANI	Üroloji
	BULUNDUĞU MERKEZ	Sultanbeyli Devlet Hastanesi
	YARDIMCI ARAŞTIRMACI UNVANI ADI SOYADI	Doç.Dr. Kadem ARSLAN
	ARAŞTIRMANIN STATÜSÜ	☐ Yüksek Lisans Tezi ☐ Doktora Tezi ☐ Tıpta Uzmanlık Tezi<br>■ Bilimsel Araştırma Projesi ☐ Diğer: ....
	ARAŞTIRMANIN FAZI VE TÜRÜ	Gözlemsel ilaç araştırmaları ☐
		Gözlemsel epidemiyolojik araştırmalar ☐
		Anket, sorgulama ve görüşme şeklinde araştırmalar ■
		İnsan biyolojik materyallerinin kullanımını içeren araştırmalar ■
		Tıbbi kayıtlar ve hasta bilgileri üzerinde yapılan araştırmalar ☐
		Tıbbi cihaz klinik araştırması ☐
		Nitel araştırmalar ☐
		Diğer ise belirtiniz
	ARAŞTIRMAYA KATILAN MERKEZLER	TEK MERKEZ ■ · ÇOK MERKEZ ☐ · ULUSAL ☐ · ULUSLARARASI ☐
	ARAŞTIRMANIN YAPILACAĞI MERKEZ	SULTANBEYLİ DEVLET HASTANESİ

Etik Kurul Başkanının
Unvanı / Adı /Soyadı:
Doç. Dr. Orhun
SİNANOĞLU imza:

Not: Etik kurul başkanı, imzasının yer almadığı her sayfaya imza atmalıdır

BİLİMSEL ARAŞTIRMALAR ETİK KURULU KARAR FORMU

ARAŞTIRMANIN AÇIK ADI	Vitamin B12 eksikliği olan prematür ejakülasyon hastalarında vitamin B12 replasmanı öncesi ve sonrası prematür ejakülasyonun değerlendirilmesi				
ETİK KURUL BAŞVURU DOSYA NO	Dosya numara : 381				
**DEĞERLENDİRİLEN BELGELER**	**Belge Adı**	**Tarih**	**Dili** : Türkçe ■ İngilizce □ Diğer □		
	ONAY YAZILARI	25.12.2024	Evet x	Hayır □	Gerek Yok □
	BAŞVURU FORMU	25.12.2024	Evet x	Hayır □	Gerek Yok □
	ANKET FORMU	25.12.2024	Evet x	Hayır □	Gerek Yok □
**DEĞERLENDİRİLEN DİĞER BELGELER**	ARAŞTIRMA BÜTÇESİ	25.12.2024	Evet x	Hayır □	Gerek Yok □
	ÖZGEÇMİŞ	25.12.2024	Evet x	Hayır □	Gerek Yok □
	BİLGİLENDİRİLMİŞ GÖNÜLLÜ OLUR FORMU	25.12.2024	Evet x	Hayır □	Gerek Yok □
	PEDİATRİK HASTALAR İÇİN ONAY	25.12.2024	Evet □	Hayır □	Gerek Yok x
	HELSİNKİ BİLDİRİSİ	25.12.2024	Evet x	Hayır □	Gerek Yok □
	TAAHHÜTNAME	25.12.2024	Evet x	Hayır □	Gerek Yok □
	LİTERATÜR	25.12.2024	Evet x	Hayır □	Gerek Yok □
	Diğer				
**KARAR BİLGİLERİ**	**Karar No:2024/381**				
	Yukarıda bilgileri verilen başvuru dosyası ile ilgili belgeler araştırmanın/çalışmanın gerekçe, amaç, yaklaşım ve yöntemleri dikkate alınarak incelenmiş ve **uygun olduğu** araştırmanın/çalışmanın başvuru dosyasında belirtilen merkezlerde gerçekleştirilmesinde etik ve **bilimsel sakınca olmadığı** toplantıya katılan etik kurul üye tam sayısının salt çoğunluğu ile karar verilmiştir.				

BİLİMSEL ARAŞTIRMALAR ETİK KURULU

**ETİK KURULUN ÇALIŞMA ESASI**	İlaç ve Biyolojik Ürünlerin Klinik Araştırmaları Hakkında Yönetmenlik, İyi Klinik Uygulamaları Kılavuzu
**BAŞKANIN UNVANI / ADI / SOYADI:**	DOÇ. DR. ORHUN SİNANOĞLU

**Unvanı/Adı/Soyadı**	**Uzmanlık Alanı**	**Kurumu**	**Araştırma ile İlişki**		**Katılım**		**KABUL**	**RED**	**İmza**
DOÇ. DR. ORHUN SİNANOĞLU **(KURUL BAŞKANI)**	ÜROLOJİ	SANCAKTEPE ŞEHİT PROF.DR. İLHAN VARANK EAH.	E□	H☑	E☑	H□	☑	□	[signature]
PROF. DR. ELİF TORUN PARMAKSIZ **(KURUL BAŞKAN YRD.)**	GÖĞÜS HASTALIKLARI	SANCAKTEPE ŞEHİT PROF.DR. İLHAN VARANK EAH.	E□	H□	E□	H□	□	□	
DOÇ.DR. HASAN HÜSEYİN MUTLU	AİLE HEKİMİ	SANCAKTEPE ŞEHİT PROF.DR. İLHAN VARANK EAH.	E□	H□	E□	H□	□	□	
DOÇ.DR. ÖZDEM ERTÜRK ÇETİN	NÖROLOJİ	SANCAKTEPE ŞEHİT PROF.DR. İLHAN VARANK EAH.	E□	H☑	E☑	H□	☑	□	[signature]
DOÇ. DR.ARZU BİLGE TEKİN	KADIN HASTALIKLARI VE DOĞUM	SANCAKTEPE ŞEHİT PROF.DR. İLHAN VARANK EAH.	E□	H☑	E☑	H□	☑	□	[signature]
UZM. DR. ŞENAY ÇOŞKUN	ÇOCUK SAĞLIĞI VE HASTALIKLARI	SANCAKTEPE ŞEHİT PROF.DR. İLHAN VARANK EAH.	E□	H☑	E☑	H□	☑	□	[signature]
UZM. DR. SULTAN GÖZDE TEMİZ	İÇ HASTALIKLARI	SANCAKTEPE ŞEHİT PROF.DR. İLHAN VARANK EAH.	E□	H☑	E☑	H□	☑	□	[signature]
DOÇ. DR. ÇİĞDEM DİCLE ARICAN	PATOLOJİ	SANCAKTEPE ŞEHİT PROF.DR. İLHAN VARANK EAH.	E□	H☑	E☑	H□	☑	□	[signature]
PROF.DR. DERYA ÖZTÜRK ENGİN	ENFEKSİYON HASTALIKLARI	SANCAKTEPE ŞEHİT PROF.DR. İLHAN VARANK EAH.	E□	H☑	E☑	H□	☑	□	[signature]
DOÇ. DR TUHAN KURTULMUŞ	ORTOPEDİ	SANCAKTEPE ŞEHİT PROF.DR. İLHAN VARANK EAH.	E□	H□	E□	H□	□	□	
PROF. DR. EMRE YALÇINKAYA	KARDİYOLOJİ	SANCAKTEPE ŞEHİT PROF.DR. İLHAN VARANK EAH.	E□	H□	E□	H□	□	□	

Etik Kurul Başkanının Unvanı / Adı /Soyadı:

Doç. Dr. Orhun SİNANOĞLU imza: [signature]

Not: Etik kurul başkanı, imzasının yer almadığı her sayfaya imza atmalıdır.
